# Cases of Vaccine and Variolous Inoculation

**Published:** 1805-06-01

**Authors:** William Forbes

**Affiliations:** Member of the Royal College of Surgeons, London


*Cases of Vaccine and Variolous Inoculation, by* William Forbes, *Member of the Royal College of Surgeons, London.*

THE reception, which Vaccine Inoculation has experienced from all ranks of society, affords the best possible proof of the public sentiment respecting the destructive influence of the small pox, which, even under the milder form of inoculation, exclusively of its leaving behind it various painful consequences, has, in many instances, proved fatal, and always been a source of contagion.

It was, however, to be expected, that many would oppose this new practice; and some have accordingly objected, that although the matter, when taken immediately from the cow, is a permanent security against the future contagion of the small pox, yet, that when the vaccine virus is communicated from one human subject to another, it loses that preventive influence.

To prove this, some cases are related, in which the small pox is said to have occurred after vaccination; and, whilst the advocates of vaccine inoculation appear unwilling to admit the possibility of the small pox succeeding, if the pustule have assumed the genuine character (although there were no apparent indisposition), the opponents have been equally anxious to establish every failurre, not as arising from adventitious circumstances only, but from a real defect in the permanent power of the vaccine virus to prevent the future contagion of the small pox.

The following case of failure of Vaccine Inoculation gives an appearance of plausibility to this opinion:

In December 1802, Stephen Brown, a young man in perfect health, was inoculated with vaccine matter, in both arms; the weather was cold, and the inflammation so slow, that some days elapsed before it could be determined whether the virus had taken effect, which it did in one arm only; the subsequent progress was likewise slow in all its stages of vesication, efflorescence, scabbing, and unaccompanied with any apparent indisposition, and after some weeks the scab came off.

With matter taken from the above patient, John Heath was inoculated. The progressive stages of the vaccine pustule were precisely the same on the arms of the latter: as were also those of two children, inoculated from this last source.

On the 18th of February, 1805, Mr. John Prior, of Clapham, informed me, that the above-mentioned Stephen Brown was then under his care with the natural small pox. On seeing him, he gave the following history: That on the 11th of February he was seized with violent pain in his head, back, and limbs, accompanied with cold shivering, alternated with heat, had lost his appetite, and was thirsty. These complaints continued until the 15th; on the morning of which day some red spots appeared, first upon his face, then upon the chest and upper extremities: afterwards they were observed upon the body and lower extremities. From their first appearance, the symptomatic fever was relieved, and the eruptions went on to maturation, but were of a mild kind, and in considerable number. It is necessary to mention here, that the small pox was then in his neighbourhood.

He gave also a distinct account of the vaccine inoculation: he remembered the progress of the pustule, and mentioned his having frequently examined the arm of John Heath (his shopmate) comparing the appearances with those that had taken place in his own, the cicatrix of which is still perfect.

Every medical gentleman who saw this man was convinced, that he had the natural small pox: as a corroborating proof, two children were inoculated with matter taken from one of the pustules, on the 19th and 20th of

February

February, both of whom had the disease entirely to the satisfaction of the several gentlemen who visited them.

The two children who had been formerly inoculated with vaccine matter, taken from John Heath, were now subjected to the variolous test by inoculation with matter taken from one of those patients. The local effect produced upon the infected arms was as complete as is usual in those cases where the constitution had previously undergone that change which secures it from the future influence of the variola.

It may be remarked, that the vaccine inoculation in Stephen Brown must have been perfect, as matter taken from his arm produced the same disease in that of John Heath, from whom the two children were inoculated, and whose arms likewise exhibited a beautiful specimen of the cow pox.

I shall subjoin a few observations to prove, that the cause of failure in the vaccine matter, in the case of Stephen Brown, is not to be attributed to a defect in the preventive power of the vaccine virus, but to the circumstance of his constitution not having undergone that change which is necessary to secure it from the future contagion of the small pox, notwithstanding the perfect appearance of the pustule upon his arm. This may be illustrated by the following case:

A child having the confluent small pox, was occasionally nursed by his brother, upon whose chin was observed a perfect pustule; he had no complaint, and never had the disease. In about fourteen days after, with the previous symptomatic fever, he had a plentiful eruption of the small pox, in a mild form.

This opinion may also be supported from the analogy that appears between the vaccine and variolous inoculations, which the subjoined cases are designed to prove.

On three children, who were inoculated with small-pox matter, the appearance of the pustule upon their arms was so complete, that, although there were no apparent indisposition, several medical gentlemen, who saw these patients, pronounced them perfectly secure from the infection of the natural small pox; yet as there appeared no symptomatic fever, the criterion of the constitution having undergone the change necessary, to prevent the future contagion, they were again inoculated with variolous matter; and while two of them resisted its general influence, their arms only being infected, the other child had the usual antecedent symptoms, followed by an eruption of small-pox pustules.

Another experiment of the same kind was made upon a child in another family, under precisely the same circumstances, and (much to their surprise) with the same effect.

Some of these children had given the small pox to others, while they themselves were afterwards subject to the disease.

It cannot admit of a doubt, but that these failures were owing to the action of the variolous matter having been confined to the infected part only, which may be called one of Nature's phenomena, and are not to be ascribed to a want of durability in the variolous matter to prevent future contagion.

In consequence of similar occurrences, many were averse to the inoculated small pox, from an apprehension of future insecurity.

There appears, however, a difference between the two inoculations: in the vaccine it is allowed, that if the pustule produced be genuine, without any perceptible complaint (as was the case with Stephen Brown) the patient is declared perfectly secure from the future contagion of the small pox.

In the variolous inoculation, the symptomatic fever forms the criterion of future security.

It does not appear to me probable, that such cases of local affection will occur oftener in the cow pox than in the small pox; at least, I feel myself warranted to draw this conclusion, from having failed to produce the small pox, either by inoculation, or exposure to the contagion, in every experiment I have hitherto made, from the period of six months to five years after vaccination.

*Camberwell, April* 15, 1805.

